# Supplementary figures and images for: A Pilot Study to Assess the Safety and Efficacy of Umbilical Cord Blood-Derived Mesenchymal Stromal Cells for the Treatment of Synovitis in Horses
Source: Animals (Basel). 2024 Nov 26;14(23):3406. doi: 10.3390/ani14233406 (PMC11640105; doi:10.3390/ani14233406)

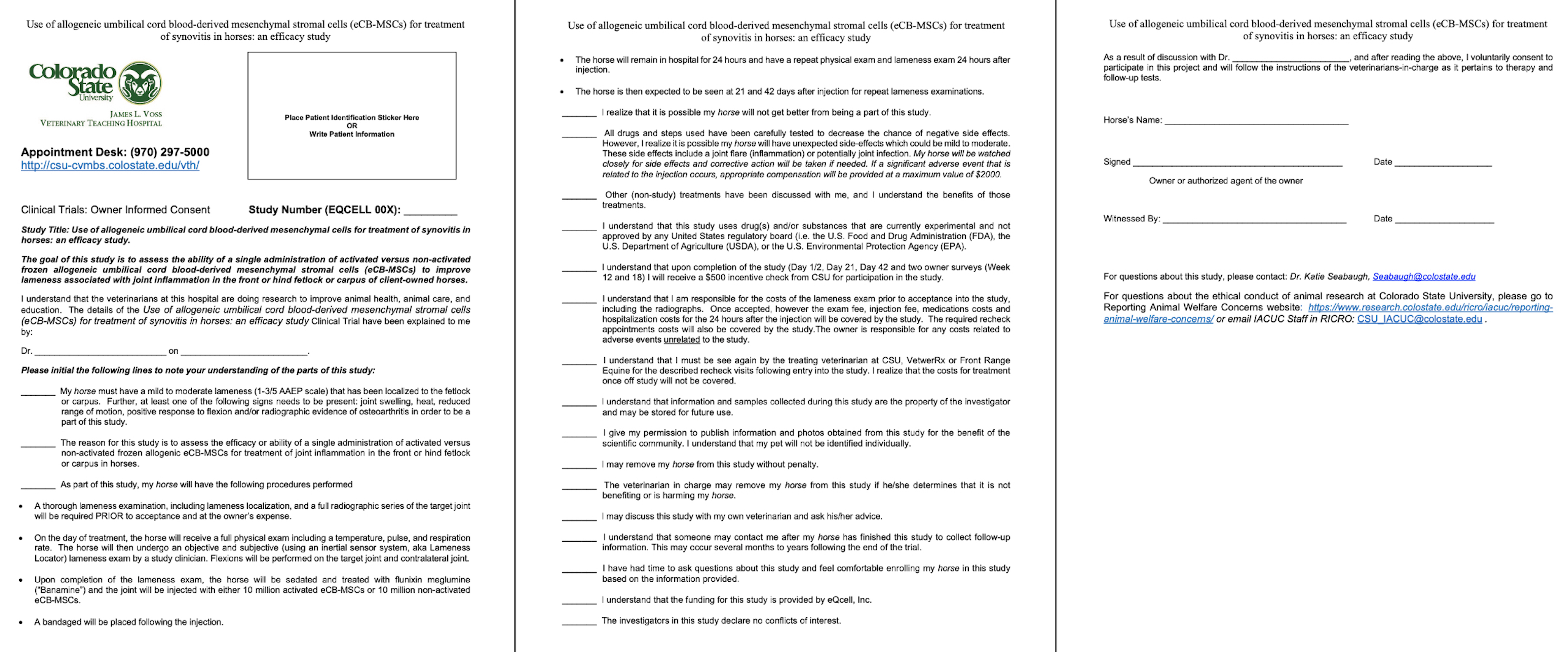

Supplement: Supplementary file 1 [file animals-14-03406-s001.zip › Figure S1 - Consent.jpg]

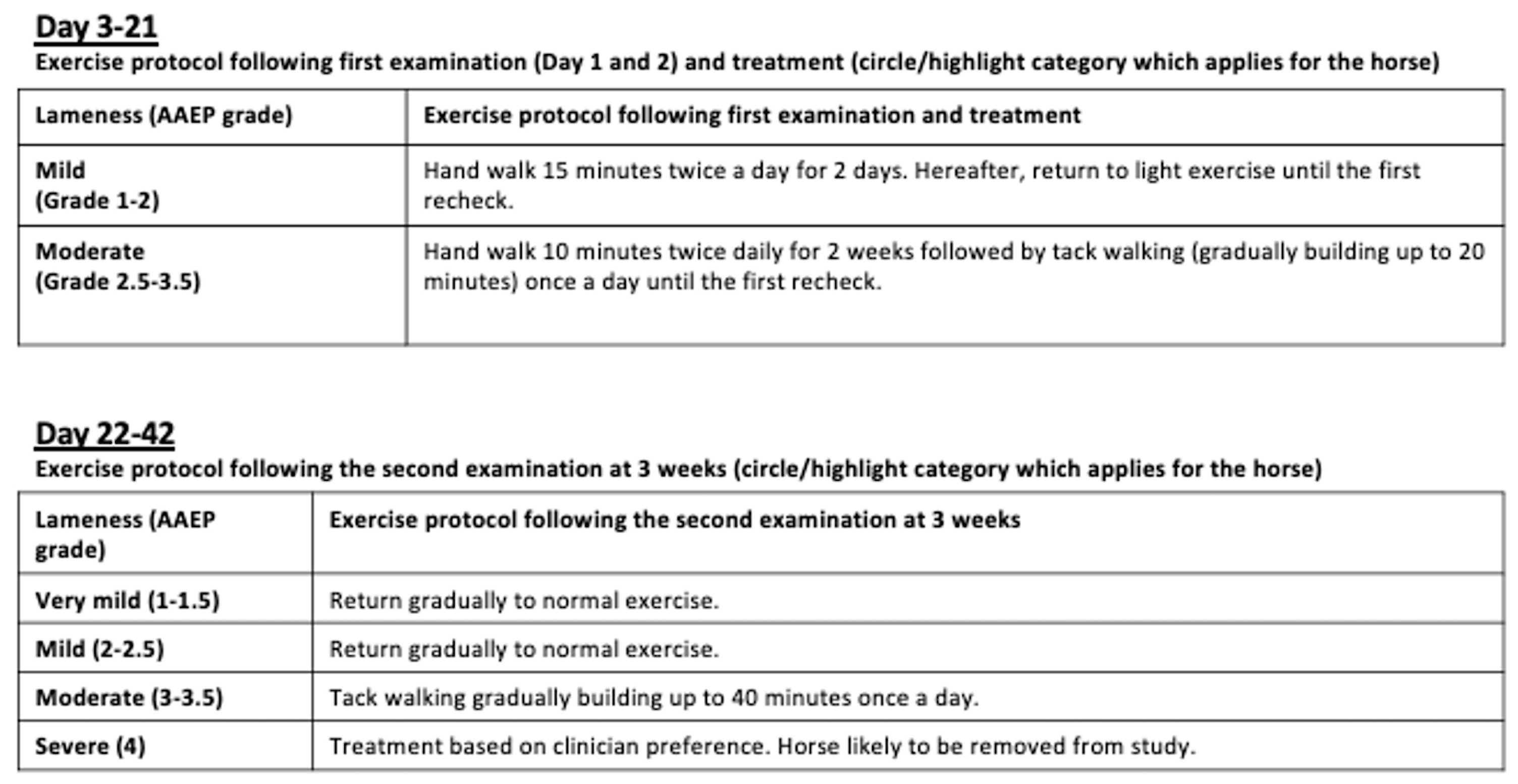

Supplement: Supplementary file 1 [file animals-14-03406-s001.zip › Figure S2 Exercise.jpg]

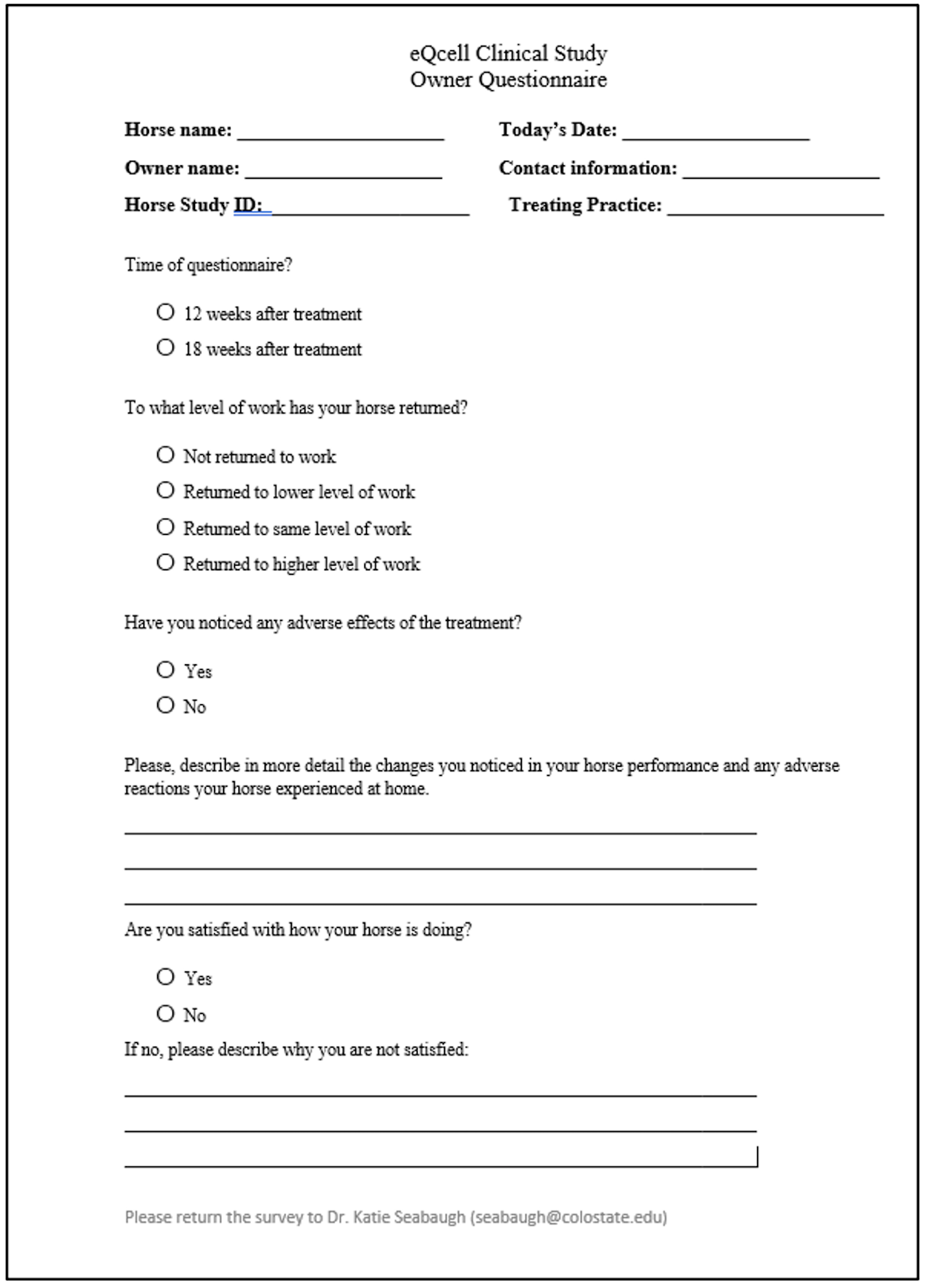

Supplement: Supplementary file 1 [file animals-14-03406-s001.zip › Figure S3 Questionnaire.jpg]

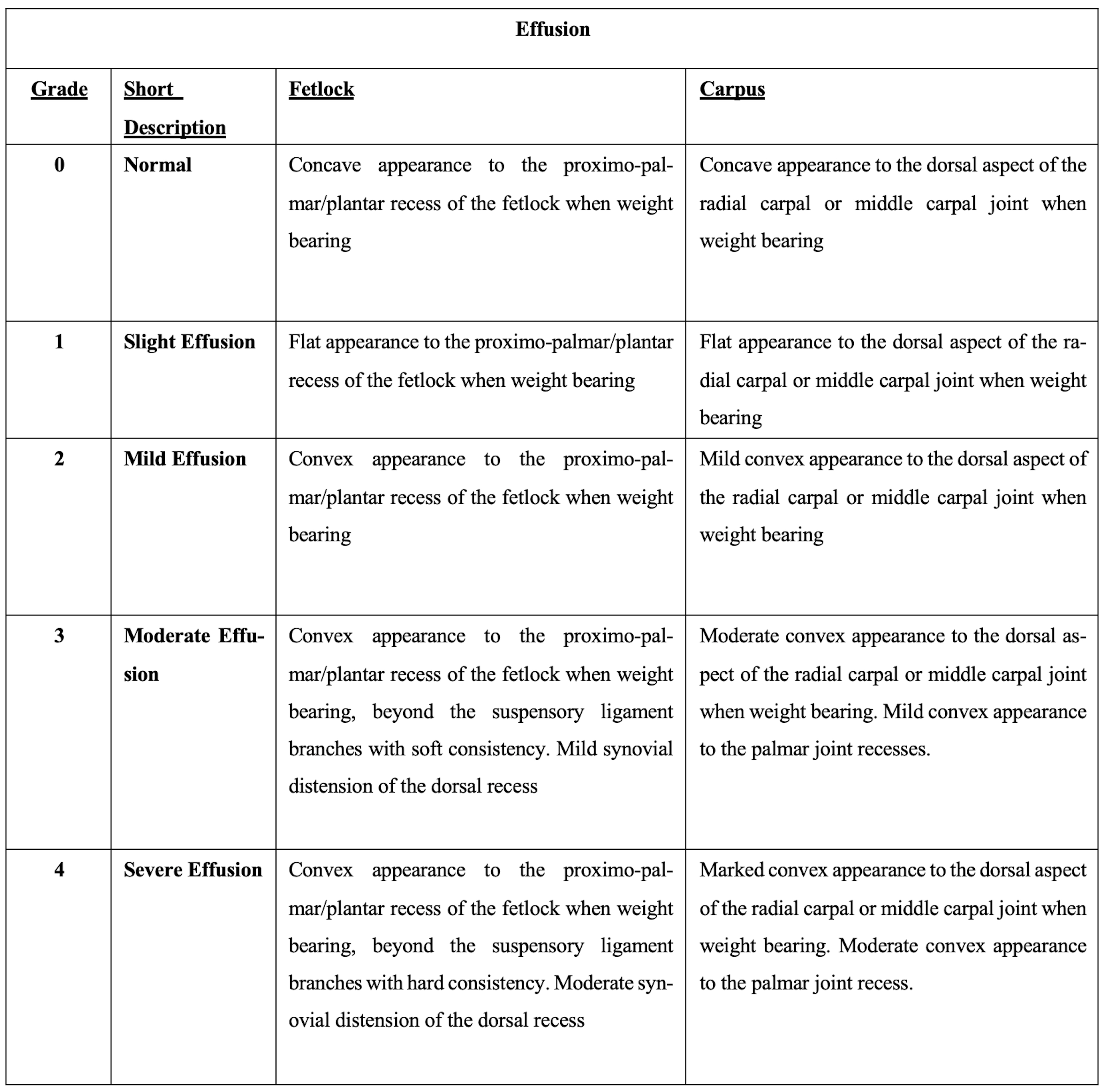

Supplement: Supplementary file 1 [file animals-14-03406-s001.zip › Table S1 effusion.jpg]

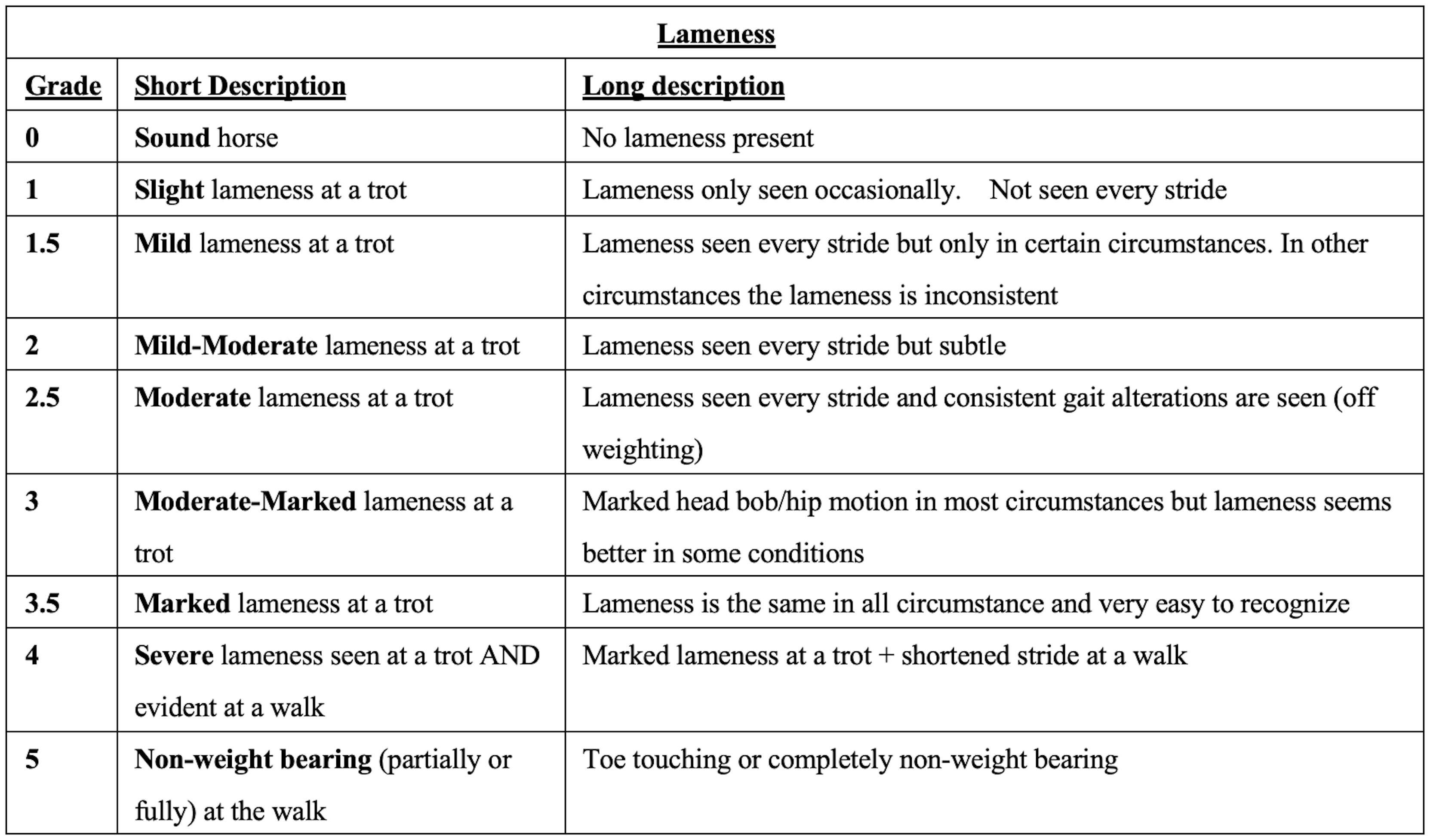

Supplement: Supplementary file 1 [file animals-14-03406-s001.zip › Table S2 lameness.jpg]

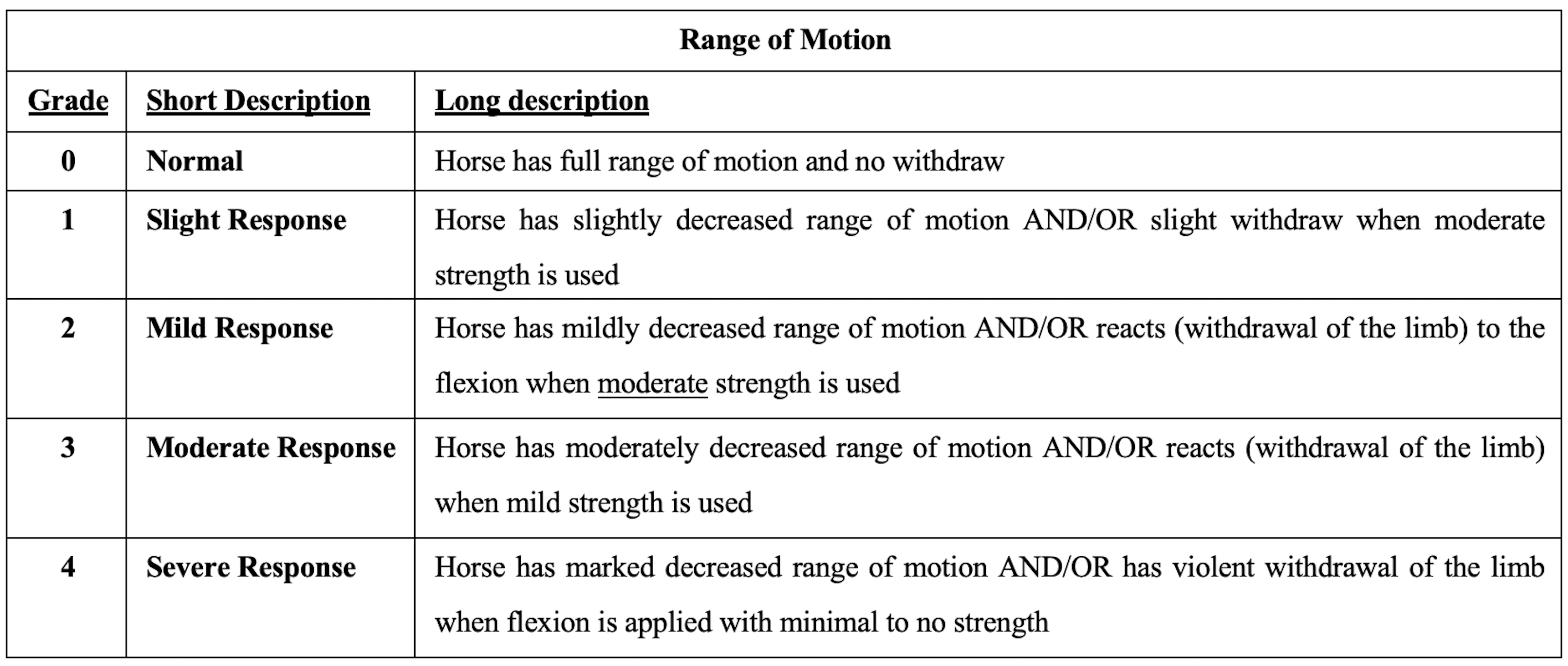

Supplement: Supplementary file 1 [file animals-14-03406-s001.zip › Table S3 ROM.jpg]

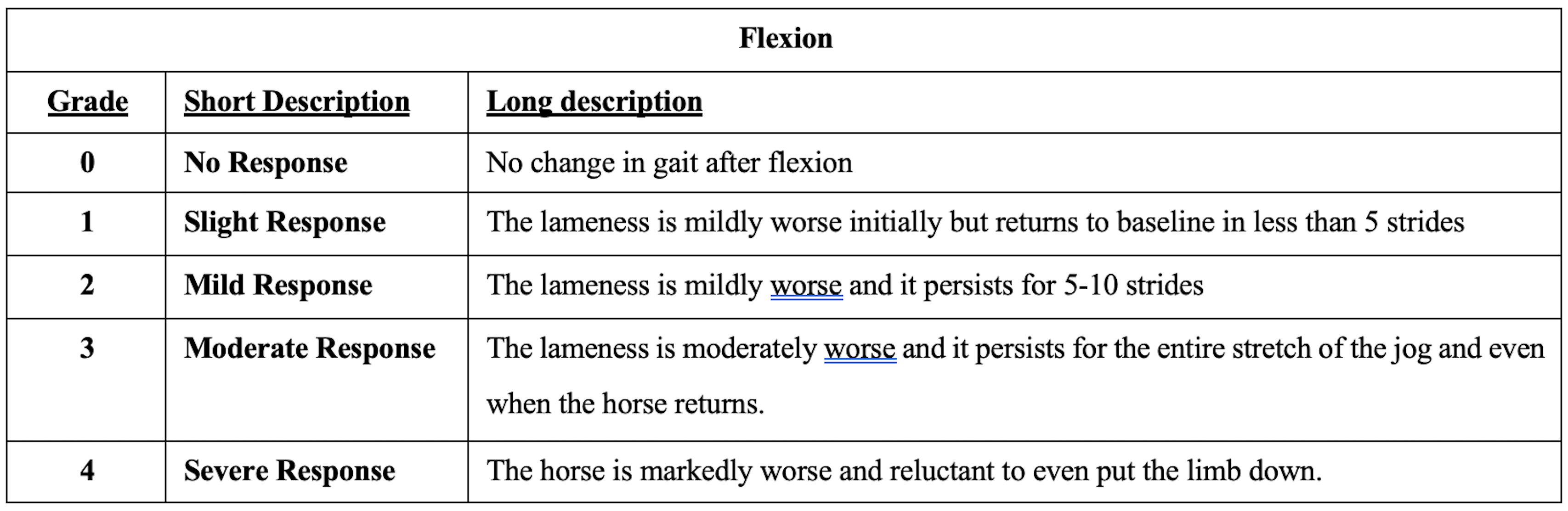

Supplement: Supplementary file 1 [file animals-14-03406-s001.zip › Table S4 Flexion.jpg]
